# Health benefits of bluefin tuna consumption: (*Thunnus thynnus*) as a case study

**DOI:** 10.3389/fnut.2024.1340121

**Published:** 2024-04-02

**Authors:** F. Chamorro, L. Cassani, P. Garcia-Oliveira, M. Barral-Martinez, A. O. S. Jorge, A. G. Pereira, Paz Otero, M. Fraga-Corral, M. Beatriz P. P. Oliveira, M. A. Prieto

**Affiliations:** ^1^Nutrition and Bromatology Group, Department of Analytical Chemistry and Food Science, Instituto de Agroecoloxía e Alimentación (IAA)-CITEXVI, Universidade de Vigo, Vigo, Spain; ^2^REQUIMTE/Serviço de Bromatologia, Faculdade de Farmácia da Universidade do Porto, Porto, Portugal; ^3^LAQV@REQUIMTE, Department of Chemical Sciences, Faculdade de Farmácia, Universidade do Porto, Porto, Portugal

**Keywords:** *Thynnus thunnus*, nutritional composition, beneficial properties, risk-benefit ratio, food safety

## Abstract

Consumers are increasingly interested in food products with high nutritional value and health benefits. For instance, fish consumption is linked with diverse positive health benefits and the prevention of certain widespread disorders, such as obesity, metabolic syndrome, or cardiovascular diseases. These benefits have been attributed to its excellent nutritional value (large amounts of high-quality fatty acids, proteins, vitamins, and minerals) and bioactive compounds, while being relatively low-caloric. Atlantic bluefin tuna (*Thunnus tynnus*) is one of the most consumed species worldwide, motivated by its good nutritional and organoleptic characteristics. Recently, some organizations have proposed limitations on its consumption due to the presence of contaminants, mainly heavy metals such as mercury. However, several studies have reported that most specimens hold lower levels of contaminants than the established limits and that their richness in selenium effectively limits the contaminants’ bioaccessibility in the human body. Considering this situation, this study aims to provide baseline data about the nutritional composition and the latest evidence regarding the beneficial effects of Atlantic bluefin tuna consumption. A review of the risk-benefit ratio was also conducted to evaluate the safety of its consumption, considering the current suggested limitations to this species’ consumption.

## 1 Introduction

Nowadays, consumers are increasingly aware of the beneficial effects on health of certain foods and the adoption of well-balanced diets. In this sense, most marine products, especially fish, are widely appealing for their high nutritional value (1). Fish consumption has been traditionally linked to many health benefits due to their high omega-3 polyunsaturated fatty acids (PUFAs) content (2), being of particular interest eicosapentaenoic acid (EPA) and docosahexaenoic acid (DHA) (3–6). Both compounds are well-known for their positive effects on the cardiovascular system and the nervous system, as well as the control of inflammatory processes in vertebrates, being beneficial in various human pathologies and disorders like obesity or metabolic syndrome (7, 8). More recently, fish proteins, peptides, and amino acids have harbored attention as they have shown properties similar to PUFAs (9). In addition, fish is also a significant source of vitamin B12 and vitamin D (10). Vitamin B12 is required to form red blood cells and DNA. Deficiency of vitamin D leads to rickets, a low bone mineral density and thereby to osteoporosis, among other pathologies. Fish is also an important source of essential minerals, like copper (Cu), manganese (Mn), zinc (Zn), and selenium (Se), which participate in many biological processes as part of numerous enzymes (10). Cu plays an important role as a catalytic cofactor in numerous critical enzyme reactions in metabolism (11). Mn deficiency results in poor reproductive performance, congenital malformations, growth retardation in offspring, and abnormal function of bone and cartilage (12). Zn is required in the stabilization of the structure of many proteins at all levels of cellular signal transduction (13). Finally, Se plays a fundamental role in reproduction, thyroid function, DNA replication and protection against microbes and oxidant compounds (14). Therefore, fish is considered one of the healthiest foods on global scale and is a fundamental part of a healthy and well-balanced diets.

However, in recent years, some national and international Food Safety Agencies, like the Spanish Agency for Consumer Affairs Food Safety and Nutrition (AECOSAN) and the US Food and Drug Administration (FDA), among others, have recommended limiting the consumption of certain species of fish in children and pregnant women (15). The reason for this limitation is the level of certain heavy metals, like mercury (Hg), found in some blue fish such as *Prionace glauca* (blue shark)*, Isurus oxyrinchus* (blue pointer or bonito shark), *Xiphias gladius* (swordfish) and *Thunnus thynnus* (Atlantic bluefin tuna) (16–18). When Hg reaches the sea from soil or chemical industry, it accumulates in marine species throughout the food chain; the larger and longer predator fish are, the higher the levels found (19, 20). Thus, large fish such as swordfish, bluefin tuna, and sharks accumulate these compounds in their tissues since they feed on small fish. Despite this, some recent studies point out that the risk of Hg intake due to fishery products consumption is not as substantial as commonly believed (21, 22). The European Food Safety Agency (EFSA) has recently stated that limiting fish consumption due to Hg’s presence can lead to more significant health risks than moderate consumption (23). In fact, the European legislation (Commission Regulation (EU) No. 1881/2006) established maximum levels of Hg in fish (0.5–1 mg/kg) based on the level of consumer exposure (24), but the majority of fishery products currently show levels much lower than the limits set in the legislation (21, 22). This points to the current limitation on seafood consumption being somewhat exaggerated. In addition, several studies show that Se, an essential mineral commonly present in seafood, may also protect against the toxic effects of Hg, mainly its most dangerous form of organic methylmercury (25, 26). Thus, the Hg: Se ratio should also be considered when assessing the risk linked to fish intake (11).

**Figure 1:**
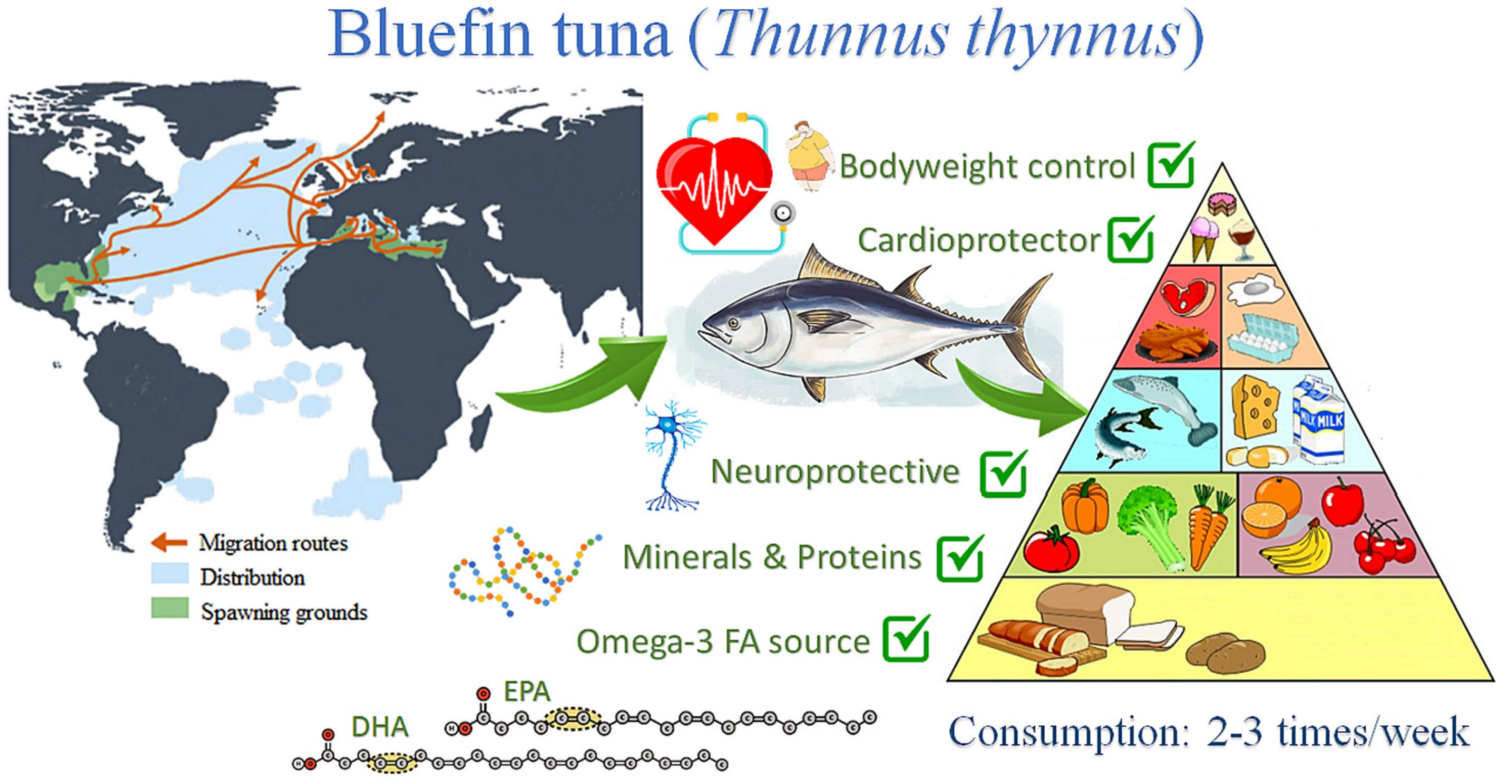
Schematic representation of bluefin tuna (*Thunnus thynnus*) distribution and their main health impact derived from their consumption.

The present study will be focused on the Atlantic bluefin tuna (ABFT) *Thunnus thynnus* (L., 1758), a top-level pelagic predator distributed throughout the Atlantic Ocean, from the Canary Islands to Ireland, with incursions to Norway and the North Sea, the Baltic, and the Barents Sea, Mediterranean and Black Sea, also in Canada and South America, along the Brazilian coast (27) (Figure 1). The species is very voracious and feeds on many other fishes, crustaceans, and cephalopods (28). The generic name of bluefin tuna incorporates three species: the ABFT *Thunnus thynnus*, the Pacific bluefin tuna *Thunnus orientalis,* and the southern bluefin tuna *Thunnus maccoyii*. Throughout history, bluefin tuna *Thunnus thynnus* has been exploited in the Mediterranean for thousands of years until the end of the 20th century (29). Research on bluefin tuna farming began in the 1970s in Japan, and numerous business initiatives for farming have been launched since then (30). Several studies have been carried out in various field of research such as reproduction, nutrition, genetics, pathology, diseases, and engineering, among others (31, 32). In addition, numerous projects have been launched to improve the captive reproduction of this species, both from the business and research sectors. In a recent study, the European Market Observatory for Fisheries and Aquaculture Products (EUMOFA) shows that tuna is Europe’s most consumed marine species, followed by cod, salmon, and Alaska pollock (33). The consumption of tuna in Europe is around 3.07 kg *per capita*, from which 99.2% is wild-caught and only 0.83% is farmed (33). There is a growing demand for fresh tuna *Thunnus thynnus* in Europe. Their production is currently limited to the Mediterranean Sea, mainly in Spain, France, Italy, and to a lesser extent, Portugal, Malta, Croatia, Cyprus, and Greece (34). There has been an essential economic contribution from the bluefin tuna fishing industry, with a value of sale of more than 875 million euros in the Mediterranean Sea since 2018 (35). However, it is necessary to improve the fisheries management to make fishing more sustainable from an environmental point of view. In this sense, the treatment and recovery of the waste originated in such an industry could reduce these environmental issues. By-products from bluefin tuna have several bioactive compounds of considerable economic value that can be extracted and obtained from this discarded biomass following the principles of the circular economy (36).

In this context, the present study is focused on the nutritional composition and contaminants of ABFT *Thunnus thynnus*, including the latest evidence on human health impact and the assessment of the risk-benefit ratio of its consumption. Knowledge about the nutritional composition and risk-benefit ratio is valuable for consumers to change their diet conscientiously according to their life cycle stages.

## 2 Nutritional composition

It is well-known that fish consumption has numerous benefits for human health (37, 38). ABFT *Thunnus thnunnus* is valued as an excellent food worldwide due to its good nutritional and sensory quality, making it a favorite choice in the seafood market. Consequently, many organizations have been interested in developing aquaculture and processing technology to increase fishing and processing efficiency (39). In this section, we will address the nutritional composition of ABFT. Different databases were consulted to provide information about the approximate composition of fish and shellfish. Among them are the global database of FAO/INFOODS, the USDA, the United States National Marine Fisheries Service, and the United Kingdom Department of Health. Table 1 shows the composition of macro and micronutrients present in 100 g of ABFT meat, which is low in calories while providing high-quality proteins and lipids, fat-soluble vitamins, and various essential elements. In addition, the consumption of this species has been linked to a series of beneficial health effects due to the presence of bioactive compounds, including bioactive peptides present in proteins and PUFAs, mainly EPA and DHA (28, 44, 45). Nevertheless, it is important to underline that the nutritional composition of any fish may vary depending on environmental factors, age, sex, maturation stage, and the migratory behavior of each species.

**Table 1:** Nutritional composition of the bluefin tuna *Thunnus thynnus* (g/100 g dry weight) (40–43).

Nutritional component		Reference daily intake	Nutritional declaration
Energy	144 kcal		
**Macronutrients (g/100 g)**			
Proteins	23	50 g	High content
Carbohydrates	0	—	—
Lipid (total)	12		Low in saturated fat High content of omega-3 fatty acids
**Vitamins (units/100 g)**			
Thiamine (B1)	0.241 mg	1.1 mg	High content
Riboflavin (B2)	0.22 mg	1.4 mg	Font
Pantothenic acid (B5)	—	6 mg	—
Pyridoxine (B6)	0.46 mg	1.4 mg	High content
Cobalamin (B12)	5 μg	2.5 μg	High content
Folate (B9)	15 μg	200 μg	—
Niacin (B3)	17.8 mg	16 mg	High content
Vitamin A (retinol)	655 μg	800 μg	High content
Vitamin D	25 μg	5 μg	High content
Vitamin C	Traces	80 mg	—
Vitamin E	1 mg	12 mg	Font
**Minerals**			
Calcium (Ca)	38 mg	800 mg	—
Iron (Fe)	1.3 mg	14 mg	—
Iodine (I)	36.7 μg	150 μg	Font
Magnesium (Mg)	28 mg	375 mg	High content
Zinc (Zn)	1.5 mg	10 mg	Font
Sodium (Na)	43 mg	≤0.12 g	Low content
Potassium (K)	40 mg	2,000 mg	—
Phosphorus (P)	200 mg	700 mg	Font
Selenium (Se)	82 μg	55 μg	High content

### 2.1 Protein and amino acid profile

According to the data compiled, the protein content in bluefin tuna is 23 g/100 g of fresh product (Table 1). Considering that the usual protein range provided by fish is between 17–23 g, we find that bluefin tuna has a higher protein content when compared to other species. Similar results have been reported in farmed and wild bluefin tuna samples (21–23 g protein) (46, 47). In 2012, the Spanish Ministry of Agriculture, Food, and Environment published a guide on nutritional declarations and health properties of food products, where ABFT was considered a high-protein food. Additionally, in its health declarations, the European Parliament stated that these proteins contribute to increasing and conserving muscle mass and maintaining bones under normal conditions (40). Experimental studies in animals have demonstrated various benefits derived from fish protein intake. These benefits include hypocholesterolemic effects attributed to the amino acid composition of fish, although the mechanism is not clear (48); antihypertensive effects due to the presence of angiotensin-converting enzyme (ACE) inhibitor peptides (49, 50); and antiatherosclerotic effects, which are attributed to the antioxidant properties of peptides and fish protein hydrolysates (44). In addition, it has also been shown that proteins can improve insulin sensitivity, prevent metabolic syndrome, and reduce the risk of type 2 diabetes (44).

Fish proteins are better quality than red meat due to their lower collagen content and better digestibility, reported to be over 90% (6, 51). The nutritional value of a protein depends on the amino acid composition (score), the content of essential amino acids, and its susceptibility to digestion (52–54). Currently, the suggested method for assessing protein quality is a chemical score, or a protein digestibility corrected amino acid score (PDCAAS) (52–54). The amino acid profile of ABFT shows a high amount of histidine, isoleucine, leucine, lysine, threonine, tryptophan, valine, phenylalanine, and methionine (6). They are considered essential amino acids since humans do not have the ability to synthesize them, and must be incorporated into the diet (Table 1). Table 1 presents the contribution of ABFT regarding the reference daily intake. Just 100 g of ABTF cover between 44% and 69% of the requirements for all essential amino acids (55–58). Due to their amino acid profile, fish proteins can also benefit health, mainly through antioxidant and anti-inflammatory effects. For example, an adequate supply of histidine through the diet provides benefits against age-related neurodegenerative and cognitive disorders, metabolic syndrome, rheumatoid arthritis, and inflammatory bowel disease (59). The three branched-chain amino acids, leucine, isoleucine, and valine, also play a fundamental role in regulating energy homeostasis, metabolism, innate and adaptive immunity, and glucose metabolism, lipid and protein synthesis. Therefore, current evidence indicates that the adequate supply of these amino acids through the diet could positively affect the parameters associated with metabolic diseases (60). Another aspect to highlight in the amino acid content of bluefin tuna is the contribution of phenylalanine and tryptophan, as both amino acids are considered natural antidepressants (61). Tryptophan is additionally vital for the correct functionality of the brain–brain axis, gut, and immune system (62).

On the other hand, the protein content is essential from an organoleptic point of view since fish species containing small amounts of protein tend to lose a considerable amount of water during cooking, which ruins the texture of the meat (47). Thus, the high protein content of this species also contributes to its good organoleptic properties.

### 2.2 Lipid content: fatty acids profile and w-3/w-6 relation

**Figure 2:**
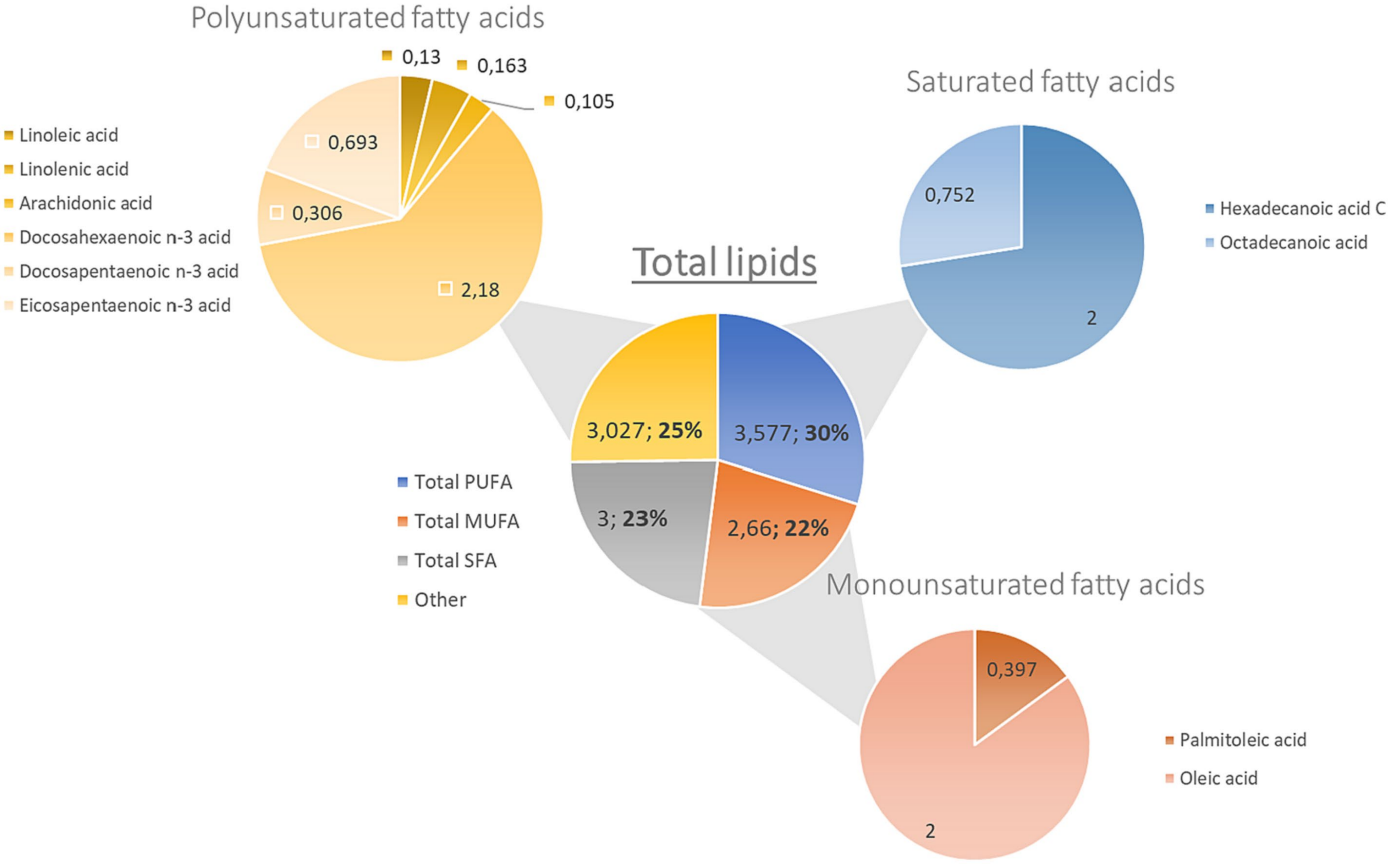
Fatty acid composition of ABPTF (g/100 g).

Lipids are macronutrients needed in the human diet and can affect health depending on the type and proportion of the dietary fatty acids consumed. It has been stated that monosaturated fatty acids (MUFAs) and PUFAs exert beneficial properties in human health (63). The lipid content of ABFT corresponds to 12 g/100 g in both wild and farmed specimens (Table 1) (64). Due to its high lipidic content, this species is considered a bluefish (64). The guidelines published in 2012 by the Spanish Ministry of Agriculture, Food and Environment declared that ABFT is low in saturated fats and high in PUFAs and that the latest contribute to the functioning of a normal heart (40). The fatty acid profile of ABFT is shown in Figure 2. PUFAs represent the main contribution to the total content of fatty acids (3.58 g/100 g) in Atlantic bluefin tuna. Within this group, DHA (2.18 g/100 g), EPA (0.693 g/100 g), and DPA (0.306 g/100 g) are the most abundant. Regarding MUFAs, oleic acid (2.263 g/100 g) and palmitic acid (0.397 g/100 g) stand out (Figure 2). Several studies have reported similar results in farmed ABFT, with 3.6 g/100 g of PUFA (47, 64). One minor difference was that the leading group of fatty acids corresponded to MUFAs, accounting for 42% of the total lipid profile (1.2 g/100 g of oleic acid and 1.1 g/100 g of erucic acid). These differences could be due to the diet received by the species in cultivation, sex, or the size of the animals under study. Other factors that may influence the lipidic composition of fish include environmental factors, age, state of maturation, and migratory behavior (41). The lipids present in ABFT have exceptional quality indices: an excellent omega 3/omega 6 ratio (9/1), an adequate polyunsaturated/saturated fatty acids ratio (1.16), and an adequate polyunsaturated/monounsaturated/saturated fatty acids ratio (2.03) (41, 47, 64). Furthermore, low levels of atherogenicity indices (AI), thrombogenicity indices (TI), and a high ratio of hypocholesterolemic to hypercholesterolemic fatty acids (HH) have been reported, indicating that the intake of this fish may exert hypocholesterolemic effects (4, 64, 65). Therefore, the consumption of ABFT could be beneficial in preventing cardiovascular diseases (66).

Various organizations such as FAO (Food and Agriculture Organization), the Academy of Nutrition and Dietetics, and the European Association for Cardiovascular recommend a minimum intake of EPA and DHA of 250 mg for adults and, in the case of pregnant and lactating women, the amount of DHA should increase between 100–200 mg (67–70). In this sense, ABFT guarantees a good quantity of fatty acids. Hundred grams of tuna meat provides 0.693 and 2.18 g of EPA and DHA, respectively, contributing to more than 100% of the reference daily intake. Consumption of these fatty acids has essential roles in human health, including promoting cardiovascular health and protection against neurological and inflammatory conditions (68, 71). Observational studies demonstrated a protective effect of fish intake on cardiovascular disease risk. In agreement, various scientific organizations affirm that the consumption of at least two servings of fish per week, where at least 1 is an oily fish, is associated with a decreased risk of death from coronary heart disease of at least 25% compared to those who do not eat fish (67–70, 72).

### 2.3 Carbohydrates

Bluefin tuna tissue comprises lipids and proteins, so the proportions of carbohydrates are minor, almost insignificant. In Table 1 it is shown that the carbohydrate content is 0 g/100 g (73).

### 2.4 Vitamins

ABFT stands out for containing significant amounts of B complex vitamins, including thiamine (B1) 0.241 mg/100 g, niacin (B3) 17.8 mg/100 g, pyridoxine (B6) 0.46 mg/100 g and cobalamin (B12) 5 μg/100 g. Thus, 100 g of bluefin tuna provides between 25% and 50% of the reference daily intake of these vitamins (Table 1). The report published by the Spanish Ministry of Agriculture, Food and Environment in 2012 established that bluefin tuna is a good source of vitamins. Within the nutritional declaration, it is also indicated that thiamine contributes to the normal functioning of energy metabolism, the nervous system, the heart, and psychological functions; niacin contributes to the maintenance of the skin and mucosa and reduces fatigue; and pyridoxine and cobalamin vitamins contribute to the normal functioning of the immune system, formation of red blood cells, and the process of cell division (40).

Additionally, bluefin tuna is rich in fat-soluble vitamins such as vitamins A, D, and E, and its consumption can contribute between 25% and 80% of the reference daily intake. Consumption of these vitamins is important because they contribute to normal iron metabolism, immune system functioning, and cell differentiation process. In the particular case of vitamin D, it contributes to the maintenance of normal bones and teeth, the maintenance of normal calcium levels in the blood, and the normal absorption and utilization of calcium and phosphorus (74–76). On the other hand, although to a lesser extent, ABFT is also a source of vitamin E, which stands out for its powerful antioxidant role and free radical scavenger (77).

### 2.5 Minerals

Minerals have a crucial role in human health and metabolism, with intake through the diet being essential (78). In this context, ABFT constitutes an excellent food source of minerals. Table 1 reports the contribution of minerals in 100 g of tuna, highlighting 28 mg of Mg, and 82 mg of Se. According to the nutritional declarations published in 2012 by the Spanish Ministry of Agriculture, Food and Environment (40), ABFT is an excellent source of these minerals. Regarding human health, Mg contributes to normal energy metabolism, electrolyte balance, normal muscle and nervous system function, normal protein synthesis, and cell division (40, 79, 80). Se is attributed to different health benefits; among them is the contribution to the normal functioning of the immune system, normal thyroid function, and the protection of cells against oxidative damage since it is part of many selenoproteins, which are responsible for biological reactions of reduction-oxidation type, antioxidant defense, metabolism of thyroid hormone and immune responses (81, 82). Furthermore, various studies report that Se can protect against environmental contaminants, such as mercury (Hg), commonly found in some fish species (83–87), but this will be discussed later (see Table 2).

**Table 2:** Amino acid profile, recommended daily intake values and percentage of contribution to the daily diet of the amino acids present in bluefin tuna (55–58).

Amino acids	Bluefin tuna (g)	Reference daily intake (g)	Input (%)
**Essentials**			
Histidine	0.687	1.14	60
Isoleucine	1.075	1.55	69
Leucine	1.896	3.43	55
Lysine	2.142	3.10	69
Methionine	0.690	1.55	44
Phenylalanine	0.911	2.69	63
Threonine	1.023	1.63	63
Tryptophan	0.261	0.41	64
Valine	1.202	1.96	61
**Semi-essentials**			
Proline	0.825	—	—
Arginine	1.396	—	—
Aspartic acid	2.388	—	—
Cystine	0.250	—	—
Glutamic acid	3.482	—	—
Glycine	1.120	—	—
Serine	0.952	—	—
Tyrosine	0.787	—	—
**Non-essentials**			
Alanine	1.411	—	—
Aspartic acid	2.388	—	—

Regarding the reference daily intake, it has been observed that 100 g of bluefin tuna can contribute 149% of Se recommendation. Additionally, ABFT also contains iodine (36.7 μg/100 g), zinc (1.5 mg/100 g), and phosphorus (200 mg/100 g), being considered as a source of these minerals (42). On the other hand, ABFT has a low contribution of sodium (43 mg/100 g), the nutritional declaration naming it as a low-content source. Thus, its consumption is attractive for low-sodium or low-salt diets, recommended, for example, to patients with hypertension.

## 3 Health benefits associated with blue fish consumption

**Figure 3:**
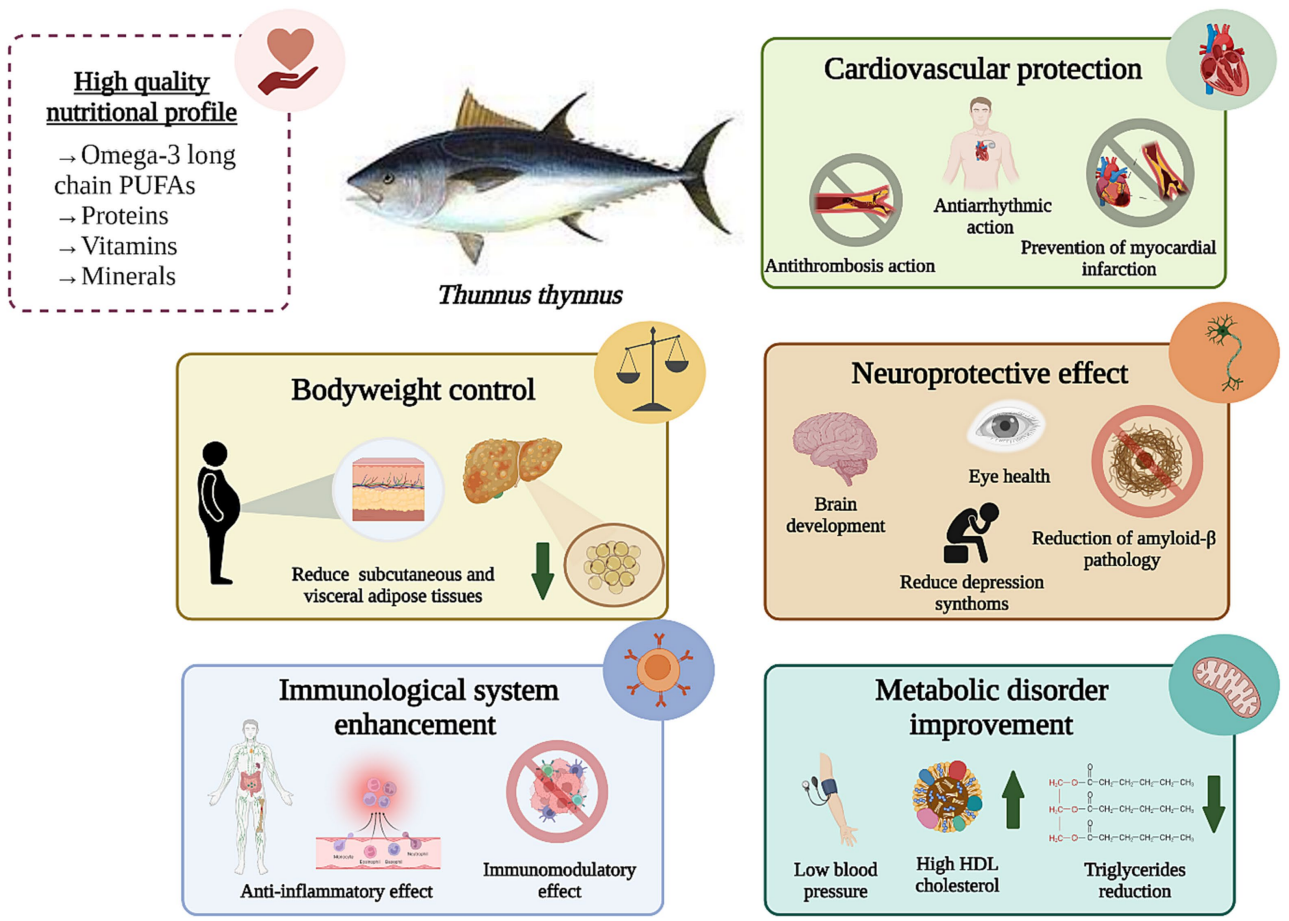
Biological activities associated with bluefin tuna consumption.

As previously mentioned, blue fish and ABFT are highly nutritious seafood products of great interest in the market and among health-conscious consumers (88). Numerous studies have linked the chemical composition of these foods with many biological properties and beneficial effects on health. These beneficial effects are mainly attributed to PUFAs, especially EPA and DHA. Additionally, fish provide other high-quality nutrients, such as proteins, vitamins, and minerals, that may have a synergic effect, reducing the incidence of certain diseases (89). The health benefits associated with fish consumption will be discussed in this section and are summarized in Table 3 and Figure 3.

**Table 3:** Different studies about omega-3 benefits in human health.

Disease type	Study	Results	Reference
Cardiovascular: stroke	Prospective cohort study Men: 43,671 Age: 40–79 years Duration: 12 years	Eating fish (*n*−3 PUFAs) once per month or more can reduce the risk of ischemic stroke in men	(90)
Cardiovascular: stroke	Prospective cohort study Women: 79,839 Age: 34–59 years Duration: 14 years	Eating fish (*n*−3 PUFAs) 2 or more times per week can reduce risk of thrombotic infarction in women	(91)
Cardiovascular and metabolic: all cancer, CVD, ischemic heart disease, ischemic and hemorrhagic strokes, and diabetes	Prospective cohort study Men: 61,127 Age: 40–74 years Duration: 12 years	Reductions of risk of total, ischemic stroke, and diabetes were 16, 37, and 39%, respectively when fish consumption is high	(92)
	Prospective cohort study Women: 73,159 Age: 40–70 years Duration: 12 years		
Obesity and overweight	Cross-sectional study People: 124 Average age: 49 Duration: not determined	There is an inverse correlation between *n*−3 PUFA and BMI, waist circumference, and hip circumference	(93)
Metabolic: type 2 diabetes	Prospective cohort study People: men (51,963) and women (64,193) Age: 40–74 (men), 40–70 (women) Duration: 12 years	There is an inverse relation between fish intake and type 2 diabetes in women. There is not a detrimental effect of fish intake in the population	(94)
Metabolic: prostate cancer	Prospective cohort study People: men (14,916) Age: 40–84 Duration: 13 years	*n*−3 PUFAs consumption can reduce the risk of prostate cancer	(95)
Cardiovascular, metabolic and obesity: body weight, cholesterol levels, inflammation	Study People: men (34) Age: 25–65 Duration: 4 weeks	*n*−3 PUFAs supplementation did not lead to a significant reduction in body weight and body fat of patients. *n*−3 PUFAs supplementation reduced triglycerides and insulin levels of patients. *n*−3 PUFAs supplementation reduced inflammatory cytokines (IL-1β, IL-6, TNF-α) in the patients	(96)

### 3.1 Cardiovascular diseases

Globally, cardiovascular diseases (CVDs) are still the leading cause of mortality. According to the World Health Organization (WHO), about 17.9 million people died in 2019 from CVDs, which represents 32% of all global deaths (97). The major risk factors that may trigger CVDs include smoking, hypertension, obesity, dyslipidemia, psycho-social stress, and unhealthy and sedentary lifestyle (98). Current first-line treatments effectively reduce CVD risk; however, adherence to healthier dietary patterns is increasingly encouraged since certain nutrients can contribute to maintaining this risk to the minimum and can be used as a preventive tool (98, 99). In this context, fish represents an important cardioprotective dietary component, attributed to its high omega-3 long chain PUFAs content, especially EPA and DHA (99). Many studies have correlated a higher fish consumption to a lower risk of CVDs, including stroke (98), coronary heart disease (99), hypertension, arrhythmias (100), and cerebrovascular disease (101). Recently, a dose-response meta-analysis showed that fish intake of 20 g/day significantly reduced total CVD mortality (4%) (102). In a further study, these authors also found a significant association between a fish intake of 15 g/day and a reduction of myocardial infarction risk by 4% (99). Increasing fish consumption to 100–700 g/week was significantly associated with stroke risk reduction by 2%–12% (103). Some differences were observed in such association between geographical regions. While a pronounced inverse relationship between fish consumption and CVDs risk was found in Asian countries, studies conducted in Western countries reported a modest U-shaped association (102). This means that both low and high fish consumption could lead to higher CVDs risk. Possibly, this variation may be attributed to different cooking fish methods employed in Asian (mainly steaming and stir-frying) and Western countries (deep-frying) being the latter more unhealthy (102).

Many biological mechanisms are responsible for the cardioprotective effects attributed to omega-3 long chain PUFAs. Among them, are anti-inflammatory (104), and antioxidant action (105), antiarrhythmic and antithrombosis action, regulation of blood lipids level, protection of vascular endothelial cells, and immune-modulatory activity (99, 100, 106).

### 3.2 Neurological diseases

Bluefish consumption has also shown beneficial neuroprotective properties attributed to omega-3 long chain PUFAs composition. These compounds have a crucial role in proper brain development, neuro transmission, neuronal differentiation and growth, gene expression, and modulation of ion channels (107, 108). It has been stated that DHA can enhance blood flow, reduce inflammation and diminish amyloid-β pathology, thus preventing a primary cognitive decline (107). In addition, DHA has vital functions in different stages of the neuronal degeneration process since this compound can keep membrane fluidity, stimulate neurotrophic factors, diminish oxidative stress and cell death and exert anti-inflammatory activities (109). By contrast, DHA levels in the brain decrease with aging, resulting in cognitive decline (108). In a meta-analysis, the impact of DHA supplementation alone or in combination with EPA on specific memory domains (working, episodic and semantic) was studied in adults. These authors found that supplementation with 1 g/day DHA/EPA significantly improved episodic memory in adults with mild memory problems, while DHA supplementation alone induced changes in semantic and working memory to a lesser extent (110).

Regarding the incorporation of fish into diet as a good source of DHA and EPA, some authors found that moderate fish consumption and supplementation with omega-3 long-chain PUFAs (0.5–1 g/day) led to a significant reduction in depression prevalence with an U-shaped association, regardless of sex, cardiometabolic disturbances or lifestyle (111). Other study reported that a decreased ratio of omega-6/omega-3 PUFAs, a reduction of omega-6 PUFAs, and increased EPA and DHA levels in Mediterranean-style diet supplemented with fish oil significantly enhanced mental health in patients with depression over 3 and 6 months. The addition of fish oil to the diet improved omega-3 PUFAs levels while reducing the omega-6 ones (112).

### 3.3 Metabolic diseases

Metabolic syndrome is a multifactorial disorder resulting from the interaction between genetic, metabolic and environmental factors that can increase the risk of suffering CVDs, type-2 diabetes and all-cause mortality (113). It has been stated that fish consumption could inversely enhance metabolic syndrome features such as insulin resistance, abdominal obesity, hypertension, and dyslipidemia since fish containing omega-3 PUFA can reduce plasma triglycerides, blood pressure, fasting blood glucose while increasing high-density lipoprotein (HDL) cholesterol (113, 114). In addition to omega-3 PUFAs, fish also contain high-quality nutrients such as vitamins, minerals, and proteins, which could contribute to reducing metabolic syndrome (113). In a cross-sectional analysis, higher fish consumption in Norwegian adults was related to a better lipid profile with high HDL cholesterol levels and reduced triglyceride content. These authors also observed that participants consuming fish once a week (aged between 60 and 70 years) showed a 36% lower risk of suffering metabolic syndrome compared to those consuming fish at a low frequency (115). Similarly, in another cross-sectional study, higher fish consumption in Iranian female adults led to a lower prevalence of metabolic syndrome features like low blood pressure and high HDL cholesterol (113).

Many biological mechanisms have been proposed to understand the beneficial effects of omega-3 PUFAs on reducing metabolic syndrome. Among them, omega-3 PUFAs may alter transcription factors activity involved in inflammatory pathways and liver lipid metabolism (116). In this way, omega-3 PUFAs may promote triglyceride oxidation in the liver, adipose tissue and skeletal muscle, thus avoiding fat accumulation in these tissues (117). In addition, omega-3 PUFAs can enhance insulin sensitivity by reducing adipose tissue inflammation and synthesizing peroxisome proliferator-activated receptor alpha (117, 118).

### 3.4 Immunological system-related diseases

The immune system protects the host from infectious agents, bacteria, and viruses. This system involves various blood-borne factors and cells (119). The phospholipids of human immune cells hold a high concentration of omega-6 PUFAs (6%–10% linoleic acid, 1%–2% dihomo-γ-linoleic and 15%–25% arachidonic acid), while low concentrations of omega-3 PUFAs (<1% α-linoleic acid, 0.1%–0.8% of EPA, and 2%–4% of DHA). The immune processes are controlled by proteins, pro-inflammatory cytokines, eicosanoids, or miscellaneous compounds (120). It has been stated that arachidonic acid is the primary precursor of eicosanoids and leads to the production of inflammatory mediators, controlling inflammatory cell activities, cytokine production, and balance within the immune system (121). Eicosanoids are a family of bioactive mediators that modulate the intensity and duration of inflammatory and immune responses. Therefore, by altering the arachidonic acid concentration, cells will have less ability to produce eicosanoids (121–123).

Some studies concluded that omega-3 long-chain PUFAs, especially EPA and DHA, could reduce immune cells’ capacity to synthesize eicosanoids from arachidonic acid. The levels of eicosanoids are widely elevated when the amount of arachidonic acid is limited (122, 124). Thus, human diets rich in fish or fish oil may increase the concentration of EPA and DHA in immune cells. The anti-inflammatory activity attributed to omega-3 PUFAs may handle their immune function. Some studies conducted in animals, mainly in rats, demonstrated that omega-3 PUFAs affected the production of inflammatory cytokines (120, 121, 125). In fact, incorporating fish oil into the diet reduced the arachidonic acid proportion while increasing EPA and DHA levels in immune cell phospholipids (126, 127). Studies carried out in humans also demonstrated the immunomodulatory effects of omega-3 PUFAs, resulting in a significant decrease in the generation of pro-inflammatory leukotriene B4 and modulating cytokine production (128–130). Studies suggested that when sufficient concentrations of fish oil are consumed, significant anti-inflammatory effects are obtained. According to some authors, 1.35–2.7 g EPA per day is the threshold intake required to achieve a significant immunological effect (131). From these results, it may be concluded that *n*−3 fatty acids can be used as therapy for any type of inflammation that involves an undesirable immune response (121). Therefore, the regular intake of ABFT may lead to a reduction in the level of inflammation and exert a crucial immunomodulatory effect.

### 3.5 Bodyweight control

Obesity is considered an energy balance disorder leading to adipose tissue dysfunction. It is associated with high levels of inflammation and metabolic abnormalities (high levels of cytokines) (132). In fact, this disorder usually appears when omega-6:omega-3 ratio is increased, and serum phospholipid *n*−3 concentrations are decreased (93). Being overweight can lead to the development of other conditions, such as insulin resistance, type 2 diabetes, and some types of CVDs (133, 134). Women have a higher prevalence of obesity and overweight than men, and it increases with age (135). In 2017, approximately 39% of the world’s adult population was overweight, and 13% were obese (136). Although there are various strategies to treat obesity and overweight, such as pharmaceuticals, surgery, or dietary supplements, the prevalence of obesity continues to rise during this decade (117). For this reason, healthy strategies to help in weight loss and reduce body fat are needed. Omega-3 PUFAs might be a good candidate to treat obesity and its related side effects due to its important role as anti-inflammatory agent (117), reducing cytokines such as IL-1, IL-6, and TNF-α (137–139).

Numerous mechanisms have been proposed to explain the effects of omega-3 PUFAs, particularly EPA and DHA, on reducing body weight and enhancing the metabolic profile, including alterations in adipose tissue gene expression, changes in adipokine release, appetite suppression, alterations in carbohydrate metabolism and increase of fat oxidation, among others (117). Despite the knowledge of these mechanisms to reduce obesity, more studies are needed to reach a conclusion. Some works have assessed the effects of omega-3 PUFAs on body weight control both in animals and humans, concluding that EPA and DHA play a key role in promoting protection against body fat gain (140–142). For instance, incorporating omega-3 PUFAs into a rat diet for 3 weeks reduced up to 30% fat weight of subcutaneous and visceral adipose tissues (142). Similarly, other authors demonstrated that obese mice fed a diet rich in omega-3 PUFAs showed a significant loss of weight (143). Other studies dealt with the effects of supplementing the diet of overweight or obese young adult men with lean fish, fatty fish or fish oil capsules during 8 weeks (144, 145). They found a significantly higher weight loss when supplemented with fish-related capsules concerning a diet without fish. On the other hand, Schulz et al. (146) found that regular fish intake led to low weight loss in men and higher weight gain in women. Another study concluded that adopting a Mediterranean diet, including a higher consumption of fish rich in omega-3 PUFAs, did not lead to significant weight changes in men and women compared with lower fish consumption (47). Nonetheless, based on clinical studies, the impact of omega-3 PUFAs on body composition is still uncertain since there is little data available to reach a conclusion.

For this reason, there is still much controversy about whether omega-3 PUFAs exert significant anti-obesity effects (90, 93, 96). In this context, despite the anti-obesity effects of omega-3 PUFAs not yet being clear, incorporating these fatty acids into the diet may mitigate weight gain or maintain weight loss (117). Moreover, they clearly play a beneficial role in obese or overweight people in contributing to reducing inflammatory cytokines levels (137–139) and inflammatory processes (117).

## 4 Importance of fish consumption during the life cycle stages

### 4.1 Recommended intake per age group

Bluefish consumption during the life cycle stages is highly relevant. Starting with pregnant women, a sufficient intake of this type of fish is not reached to meet the recommended contributions, it can generate malformations in the fetus and defects in the neural tube. In fact, the EFSA recommends the consumption of blue fish because it can be positive in avoiding cardiovascular diseases. In the first 6 months of life and even in young children, insufficient consumption of blue fish can affect their cognitive development, causing adverse effects on brain and immune function (147, 148). However, there is still no specific information or data on the optimal amounts of ABFT in pregnant women and children under 3 years (149). In children between three and 12 years, the recommended consumption is between 50 g per week, with a total of 120 g per month (150). In adults, according to EFSA, the recommended intake is 125 g per week (148).

As mentioned, ABFT provides vitamins and minerals that stand out in its nutritional composition. ABFT is a source of vitamins B (B6, B3 and B12), D, and minerals such as phosphorus or selenium, which are high contents. For instance, one serving of tuna provides 250% of the recommended intake of vitamin D (151). Table 4 shows the nutritional contribution for each portion of 100 g of ABFT as well as the recommended daily intakes for different groups of age and also differentiated by sex. For instance, every portion of 100 g of this species provides 23 g of protein, which nearly accounts for half of the recommended daily intake. Similarly, a portion of ABFT contributes to fulfilling the recommended intake of minerals, as 100 g of ABFT provides 82 mg of Se (Table 4).

**Table 4:** Nutrition offered by 100 g of bluefin tuna *Thunnus thynnus* and the recommended daily value of certain nutrients to several different targeted populations (40–43, 152, 153).

Nutrients	(g/100 g DW)	Children 7–11 months	Children 1–17 years	Pregnant women	Female >18 years	Male >18 years
**Nutrients (g/day)**						
Proteins	23	1.12 g/kg bw per day	0.67–0.85 g/kg bw per day	0.52–28 g/kg bw per day	0.66–0.83 g/kg bw per day	0.66–0.83 g/kg bw per day
Carbohydrates	—	NA	45–60 E%	ND	45–60 E%	45–60 E%
**Minerals (mg/day)**						
Ca	38	280 mg/day	390 mg/day	750 mg/day	750 mg/day	
Fe	1.3	8 mg/day	5–13 mg/day	7–16 mg/day	7–16 mg/day	6–11 mg/day
I	0.04	70 μg/day	90–500 μg/day	200 μg/day	150 μg/day	
Mn	28	0.02–0.5 mg/day	0.5–3.0 mg/day	3 mg/day	3 mg/day	
Zn	1.5	2.4 mg/day	3.6–14.2 mg/day	1.3 mg/day	6.2 mg/day	7.5–16.3 mg/day
Na	43	NA	1.1–2 g/day^*^	2 g/day^*^	2 g/day	
K	40	750 mg/day	800–3,500 mg/day	3,500 mg/day	3,500 mg/day	
P	200	160 mg/day	250 mg/day	550 mg/day	550 mg/day	
Se	0.08	15 μg/day	15 μg/day	70 μg/day	70 μg/day	
**Vitamins (mg/day)**						
A	0.6	250 μg of retinol per day	205 μg of retinol per day	540 μg of retinol per day	490 μg of retinol per day	570 μg of retinol per day
B_6_	0.46	0.6 mg/day	0.5 mg/day	1.5 mg/day	1.3–1.6 mg/day	1.5–1.7 mg/day
B_12_	0.005	1.5 μg/day	1.5–4.0 μg/day	4.5 μg/day	4 μg/day	
C	Traces	20 mg/day	15 mg/day	105 mg/day	80–95 mg/day	90–110 mg/day
D	0.025	10 μg/day	15 μg/day	15 μg/day	15 μg/day	
E	1	5 mg/day	6 mg/day	11 mg/day	11 mg/day	13 mg/day

^*^Do not consume any other fish in this category in the same week.

It is important to note that the ingestion of toxic elements studied in different investigations from samples obtained from tuna do not pose any risk to the consumers health. However, regular, or excessive consumption of tuna species could exceed the recommended weekly intake or the lower confidence limit of the reference dose, which does not necessarily pose a significant risk to consumers (149).

### 4.2 Risk-benefit ratio: toxicological assessment

EFSA has provided risk-benefit assessments of fish consumption based on scientific resources that expose the beneficial effects of fish intake and the possible risks associated with some contaminants such as Hg or methylmercury (MeHg) (23, 150, 154, 155). In this sense, in 2012 EFSA updated the tolerable weekly intake (TWI) of MeHg, establishing the limit at 1.3 μg/kg of body weight and for inorganic Hg 4 μg/kg of body weight (150). These limits were adopted based on the assessment of different outcomes. Among them, several biomarkers were used to provide precise data for MeHg exposure such as red blood cells, hair, toenail, or fingernail whereas plasma and urine samples were preferred for Hg. Data obtained from *in vivo* assays based on different experimental animals and epidemiological studies from the Faroe Islands and Seychelles such as the Hg and MeHg toxicity in prenatal neurodevelopment, were also used as reference. To assess dietary exposure, it was assumed that the total content of Hg in fish was 100% as MeHg and a bioavailability in the body of 100%. Subsequently, EFSA made a scientific statement where panel members addressed the benefits of fish consumption, such as those due to the PUFAs content and its capacity to counteract to the risks of MeHg. Considering all this data and factors, EFSA concluded that an intake of 1 to 4 servings per week of fish was associated with beneficial effects in adults with coronary artery disease. In this range of fish consumption, health benefits outweigh risks, especially compared to people who do not consume fish (23). In addition, the EFSA stated that this frequency of consumption (1–4 servings/week) has been associated with a lower risk of mortality from coronary heart disease in adults and is compatible with current intakes and recommendations in most European countries. This statement refers to fish *per se* and considers the beneficial and adverse effects of nutrients and non-nutrients, including contaminants such as MeHg, which may be present in fish (23). However, in the risk assessment, EFSA considers children under 10 years of age and women during pregnancy, lactation or expecting to get pregnant as sensitive populations to exposure of high levels of Hg or MeHg. Therefore, for these groups, the consumption of fish species with lower amounts of these contaminants is recommended (155). Indeed, various national food safety agencies have issued recommendations to limit the consumption of certain types of fishery products in these susceptible populations. For instance, AESAN recommends avoiding the consumption of swordfish, shark, bluefin tuna, and pike by these previously mentioned susceptible populations (156).

Various authors have pointed out that the risk-benefit assessment should consider the apparent protective effect of some nutrients such as PUFAs and Se against Hg and MeHg (83, 84, 87, 88, 157–159). Regarding the protective effect of PUFAs, DHA seems to protect against oxidative stress induced by MeHg in neuronal cells (160–162). In this sense, a study evaluated the dose-response between maternal fish consumption and the child’s verbal intelligence quotient (IQ). It was found that a maternal intake of 100 mg of DHA per day may prompt a gain of 2.8 points of verbal IQ in 18 months-old children (163). Similarly, other works reported that the continuous consumption of fish by pregnant women led to a laxer relationship between intrauterine exposure to MeHg and children’s IQ (164, 165). In accordance with the Scientific Opinion of EFSA regarding the risks for public health related to the presence of Hg and MeHg, omega 3-LC PUFAs, can counteract the negative effects of exposure to MeHg (150). In this line, the most studied nutrient for protection against MeHg appears to be Se. The bound affinity of Hg and Se is a million times greater than for sulfur in analogous forms. Indeed, several attempts have been made to design products with Hg-detox capacity using Se (e.g., Hg selenide). Possible protective modes of action of Se against MeHg toxicity include antioxidant effects, increased glutathione peroxidase activity, glutathione synthesis, elevated selenoprotein levels, and increased MeHg demethylation (157, 166). In this sense, it is suggested that a molar excess of Se compared to Hg can protect against its toxic effects. This could explain why studies of maternal populations exposed to foods that contain Hg in a molar excess of Se, such as pilot whale meat, have found adverse results in children, while populations exposed to Hg but showing a constant pattern of consumption of sea fish rich in Se showed lesser or none adverse effects (167). Subsequently, a new criterion was proposed to assess the risks of Hg exposure, the Se Health Benefit Value (HBV_Se_), which simultaneously evaluates Hg exposures and dietary Se intakes, particularly regarding Se consumption during pregnancy (157). Another risk assessment proposal is the benefit-risk value (BR_V_), this equation attempts to reflect either excess Hg or excess Se, in which case it can be assessed with respect to adequate Se intake. Various studies have shown that benefits outweigh risks when it comes to bluefin tuna consumption, as the molar ratio of Se:Hg oscillates between 1.3 and 20 and always implies a molar excess of Se compared to Hg (Table 5). In addition, HBV_Se_ values are reported to oscillate between 7.9 and 296 (Table 5); therefore, it is likely that the high Se content against Hg prevents the toxicity induced by Hg (88, 176, 178, 179).

**Table 5:** Comparison of Hg and Se concentrations (mg kg − 1 w.w.) and relation molar ratios Se/Hg and HBVSe in farmed or wild *Thunnus* sp. samples.

Species	Sampling area	Typology	Hg	Se	Se/Hg	HBV_Se_	Reference
*T. thynnus*	Malta	Farm	0.61	1.07	5.48	(−7.9 to 46.8)^*^	(88)
	Sardinia	Wild	1.68	0.64	1.32	(−59.9 to 10.7)^*^	
	Spain	Wild	0.38	NM	NM	NM	(168)
			0.21				(88)
	Italy	Wild	0.45	0.607	NM	NM	(149)
			0.25	0.73			(169)
		Farm	0.66	NM	NM	NM	(170)
	Turkey	Wild	0.45	1.05	5.49	NM	(171)
	Slovenia	Wild	0.60	0.75	NM	NM	(172)
	New Jersey	Wild	0.52	0.43	2.07	NM	(173)
	Black Sea	Wild	0.62	1.29	NM	NM	(174)
	Arabian Sea		0.08	NM			
	Medit. Sea		0.20	NM			
	Central Pacific Ocean	Wild	0.50	0.88	5.26	10.4	(88)
*T. albacares*	Taiwan	Wild	0.65	0.75	2.93	NM	(175)
		Farm	0.16	0.96	15.57	296	(176)
	Hawaii	Wild	0.30	1.25	14.1	201.7	(177)
	Mozambique	Wild	0.13	1.24	NM	NM	(174)
	Reunion Island	Wild	0.30	1.65			
	Mexico	Wild	0.16	0.53	10.3	64.5	(178)
	Spain	Farm	0.76	1.24	4.50	82.7	(14)
*T. alalunga*	Japan	Wild	0.23	1.51	20	NM	(179)
	Hawaii	Wild	0.50	0.88	5.26	45.6	(177)

Hg, mercury; Se, selenium; HBVSe, selenium health benefit value; Se/Hg, ratio molar selenium/mercury; w.w, wet weight; NM, not measured. ^*^(Min–Max).

On the other hand, some authors suggest considering the bioaccessible fraction of Se and Hg to provide a more accurate risk assessment (180–182). In this line *in vitro* gastrointestinal digestion techniques provide valuable data about the bioaccessibility of Hg and MeHg which can get decreased after cooking to around half of the original concentration (181). This change in bioaccessibility has been attributed to the effect of the temperature in the structural conformation of fish muscle proteins, which may cause loss of native protein structure. These alterations could prevent the access of the enzymes used in *in vitro* gastrointestinal digestion models to the structures to which Hg is bound such as thiol groups (181). In agreement with these outcomes, another work also found up to 40% reductions in the bioaccessible fraction of Hg in fish after cooking it (183). Therefore, for a more accurate risk assessment, all the criteria mentioned above must be considered Nevertheless, further research in the area is necessary to study the synergistic effects between the different variables, to improve the understanding of the repercussions on health regarding the intake of fish and shellfish.

## 5 Conclusion

Atlantic bluefin tuna, *Thunnus thynnus*, is a highly nutritious species rich in high-quality proteins, lipids, fat-soluble vitamins, and various essential elements essential for the proper functioning of the body. Among the nutritional composition, bioactive peptides and the omega-3-polyunsatturated fatty acids EPA and DHA have been linked to beneficial effects. In this sense, several population studies have reported the positive effects of fish consumption on human health, including protection against cardiovascular, neurological, metabolic, and immune diseases and body weight regulation. Besides, consuming this species helps achieve the intake recommendations of several vitamins and minerals. However, some limitations for some vulnerable population groups, such as young children and pregnant women, should be considered due to the presence of contaminants, especially mercury and methylmercury. However, several authors have pointed to high selenium levels’ capacity to counteract the negative effects of these contaminants. Selenium has been suggested to form complexes that reduce the bioaccessibility of mercury and methylmercury and so it would decrease their harmful effects. In this sense, some studies have evaluated this species’ risk-benefit ratio, showing a minimal risk in most cases. Nevertheless, further research and assessments of the risk of tuna consumption is still necessary to provide reliable data and help safeguard the health of humans, especially about the bioaccessibility of heavy metals, toxicity of selenium complexes or deeper evaluation of risk-benefits and exposure. These outcomes would reinforce and increase the current knowledge about Atlantic bluefin tuna consumption safety and try to define more accurate consumption recommendations.

## Author contributions

FC: Methodology, Supervision, Validation, Conceptualization, Data curation, Investigation, Writing – original draft, Writing – review & editing. LC: Investigation, Writing – review & editing, Supervision. PG-O: Conceptualization, Methodology, Project administration, Supervision, Validation, Writing – original draft. MB-M: Investigation, Writing – original draft. AJ: Conceptualization, Investigation, Methodology, Writing – original draft. AP: Conceptualization, Investigation, Methodology, Writing – original draft. PO: Writing – original draft, Writing – review & editing. MF-C: Methodology, Supervision, Writing – review & editing. MAP: Writing – review & editing, Supervision. MP: Formal analysis, Funding acquisition, Methodology, Project administration, Resources, Supervision, Validation, Visualization, Writing – review & editing.
